# Graphene and Perovskite-Based Nanocomposite for Both Electrochemical and Gas Sensor Applications: An Overview

**DOI:** 10.3390/s20236755

**Published:** 2020-11-26

**Authors:** Tse-Wei Chen, Rasu Ramachandran, Shen-Ming Chen, Ganesan Anushya, Kumarasamy Ramachandran

**Affiliations:** 1 Department of Materials, Imperial College London, London SW7 2AZ, UK; t.chen19@imperial.ac.uk; 2 Department of Chemistry, The Madura College, Vidya Nagar, Madurai 625 011, India; ramachandran@maduracollege.edu.in; 3 Electroanalysis and Bioelectrochemistry Lab, Department of Chemical Engineering and Biotechnology, National Taipei, University of Technology, No.1, Section 3, Chung-Hsiao East Road, Taipei 106, Taiwan; 4 Department of Physics, S.A.V Sahaya Thai Arts and Science (Women) College, Sahayam Nagar, Kumarapuram Road, Vadakkankulam, Tirunelveli 627 116, India; anushya@savsahayathaicollege.com; 5 Key Laboratory of Zhenjiang, Institute for Energy Research, Jiangsu University, Zhenjiang 212013, China; ram@ujs.edu.cn

**Keywords:** perovskite, graphene oxide, sensors, electrochemical sensors, detection limit, electrode stability

## Abstract

Perovskite and graphene-based nanocomposites have attracted much attention and been proven as promising candidates for both gas (H_2_S and NH_3_) and electrochemical (H_2_O_2_, CH_3_OH and glucose) sensor applications. In this review, the development of portable sensor devices on the sensitivity, selectivity, cost effectiveness, and electrode stability of chemical and electrochemical applications is summarized. The authors are mainly focused on the common analytes in gas sensors such as hydrogen sulfide, ammonia, and electrochemical sensors including non-enzymatic glucose, hydrazine, dopamine, and hydrogen peroxide. Finally, the article also addressed the stability of composite performance and outlined recent strategies for future sensor perspectives.

## 1. Introduction

**Scheme 1:**
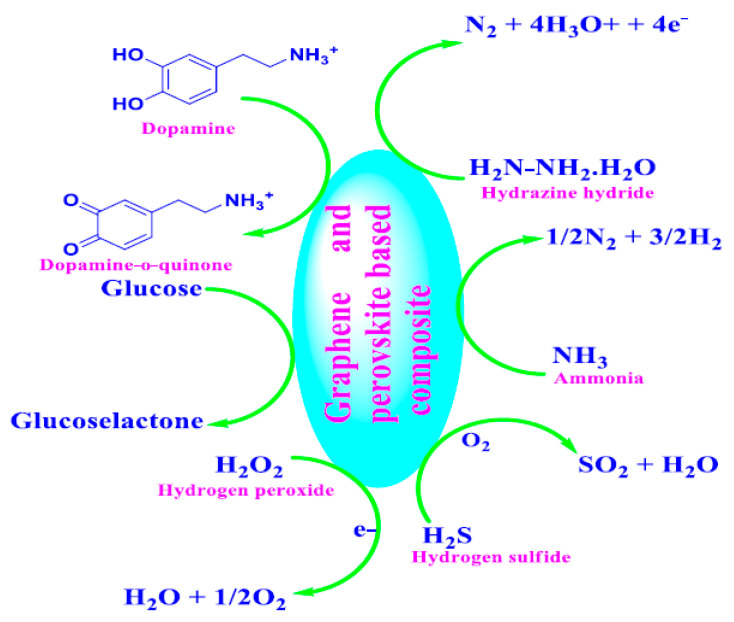
Graphene and perovskite composites for sensor applications.

The development of low cost, larger surface-to-volume ratio, low resistivity, high sensitivity, and eco-friendly natured perovskite and graphene-based composites has drawn considerable attention in both chemical [1,2] and electrochemical [3,4], sensor applications (Scheme 1). Compounds that have an ABX_3_ formula type with cations of various sizes “A” and “B” attached to anion X are known as perovskite. These perovskites are divided into three categories: perovskites of inorganic oxide, perovskites of alkaline metal halide, and perovskites of organic metal with oxide or halide anions. Furthermore, they can be synthesized from zero- to three-dimensional nanostructures and utilized in many sustainable applications. Perovskites with general formula ABO_3_ show good thermal stability with a 3–4 eV band gap and were utilized in a number of gas sensing studies. Many nanomaterial-based perovskite devices have been shown to have remarkable sensing ability for different chemical and biological species in both solid and solution states. Specifically, perovskite nanomaterials can detect small molecules such as O_2_, NO_2_, CO_2_, etc. [5]. At present, graphene oxide (GO) has received a great deal of attention in the development of nanoscience and nanotechnology, as prepared by Hummer’s method [6]. Graphene is a carbon allotrope that can be arranged in a 2D layered structure with unique properties (mechanical, thermal, and electrical) and fascinated by the modern scientific community [7]. It has a wide surface area that can be further tuned by the generation of porous structured graphene and the introduction of doping. In addition, graphene as a strongly conjugated network (π-conjugation) has hydrophobic properties that offer an ideal medium for easy immobilization of organic and inorganic molecules. Graphene also exhibits high conductivity and mediates electron transport mainly on its edge planes. These properties have created potential interest in graphene for electrochemical and biosensing-oriented applications [8]. Gas sensing, as a standard technology of intelligent systems, has recently gained growing attention in both industry and academia. Gas sensor technology has become more important due to its extensive and popular uses in the following areas; (1) agricultural processing (e.g., mine detection of methane), the automobile industry (e.g., vehicle pollution detection), medical applications (e.g., human olfactory device simulation of electronic noses), supervision of indoor air safety (e.g., carbon monoxide detection), and environmental research (e.g., greenhouse gas monitoring) [9].The composites based on perovskite and graphene have been reviewed and hold great promise for chemical as well as electrochemical applications [10,11]. Recently, the most commonly used electrochemical sensors have been significantly improved for the study of high potential, low-cost, simple, and sensitive electroactive molecules. For the sensing of urea with good sensitivity (limit of detection value of 3.79 μM) and good electrode stability during the electrochemical process, the modified glassy carbon electrode (NiS/GO/MGCE) in the case of nickel sulfide supported graphene oxide was extensively notorious [12]. Luo et al. [13] used a novel and stable copper-modified graphene sheet electrode by a potentiostatic method for rapid amperometric glucose sensing, i.e., the reported LOD value of 0.5 μM with a wide linear range of glucose value is 4.5 mM. Meanwhile, the novel acicular manganese dioxide-based graphene nanosheet (MnO_2_-GNSs) composite is a promising potential candidate with high electrocatalytic activity for the practical application of the H_2_O_2_ sensor [14]. Hydrogen peroxide (H_2_O_2_) is a simple and nonplanar molecule most commonly used in diverse fields such as biology, chemistry, environmental production. and food safety measurement [15]. In particular, graphene/nafion/azure I/Au nanoparticle-supported nanocomposites have been widely used for rapid and accurate H_2_O_2_ sensing due to unique catalytic properties, low-cost preparation, and appropriate biocompatibility. Furthermore, the composite showed a low-level detection limit value (10 μM), good reproducibility, and long durability [16].

A facile copper supported organic metal framework electrode, which is incorporated by electrochemical method with reduced graphene oxide. The fabricated nanocomposite-based graphene oxide (Cu-MOF/ERGO/ITO) was successfully used for the detection of H_2_O_2_ [17]. Zai’s group [18] successfully synthesized ternary-based reduced graphene oxide (Pt-titanium dioxide nanotubes arrays/reduced graphene oxide) using the Hummer method. The as-fabricated ternary complex has been successfully applied to the study of electrocatalytic activity with good electrode durability for methanol oxidation under alkaline conditions. A novel three-dimensional structured double hydroxide (NiCr-LDH) hybrid nanocomposite has emerged as a promising electrode material for high performance methanol oxidation [19]. Liu et al. [20] synthesized Zn_2_SnO_4_ nanoparticles decorated with reduced graphene oxide (ZTO/RGO) nanocomposite via solvothermal method and establish that the decorated 8ZTO/RGO showed high performance and good ethanol sensitivity. The gas sensor is a sensing device which can be used to detect the presence of gases surrounding the atmosphere or the environment. Zhang et al. [21] synthesized polymerized SnO_2_/rGO/PANI ternary nanocomposite via in situ polymerization technique; it also acts as an excellent candidate for the study of H_2_S gas.

In this review, electrochemical sensors such as glucose, hydrazine, dopamine, and hydrogen peroxide, as well as gas sensors such as ammonia and hydrogen sulfide sensors, have been highlighted because of their significance in environmental control, agricultural and medical applications. Various sensing parameters such as sensitivity, response speed and stability that influence the performance of both electrochemical and gas sensors are summarized.

Cui et al. [22] synthesized a unique structured and crystalline flower-like Bi_2_MoO_6_ microsphere, which exhibited excellent H_2_S gas sensing performance at a low-level detection limit of 0.1 ppb. The one-pot polylol method was used for the fabrication of palladium nanoparticles based on TiO_2_ micro-rod supported reduced graphene oxide (PdNPs/TiO_2_MRs/RGO) ternary film composite, which can also be used for the monitoring of NH_3_ sensors with long-term cyclic stability [23]. Various fabricated electrodes, diverse processing procedures and various detection techniques are of considerable value for the sensing of electrochemical and gas sensor studies (Table 1).

## 2. Electrochemical Sensors

Electrochemical sensors have gained attention because they are facile; have fast detection, inexpensive; and have simple fabrication, movability, easy miniaturization, and reliability. Electrochemical sensing methods are significant aspect for the monitoring of environmentally emerging analytes at low concentration with low detection limit (LOD), and high sensitivity over other methods. During the electrochemical detection process, the analyte can adsorb over the modified/unmodified working electrodes in the electrolyte solution. The accumulated analyte is oxidized/reduced at a particular potential. Chronoamperometry is a well-known amperometric method widely used for electrochemical detection. In this technique, the pulse potential is applied to the operating electrode and the current passing through the cell is determined by time [24]. The recognition element, electrochemical transducer, and signal processing unit for the recording, amplification, and user-friendly data representation are the main components of an electrochemical biosensor. The recognition element, the primary component of a biosensor, enables the sensor to react selectively to a single analyte from a wide number of other substances. Based on the type of recognition element, biosensors may be categorized as enzymatic, non-enzymatic, whole-cell, and immunosensors. These enzymatic biosensors are highly selective, adaptive, swift, and reversible. Glucose biosensor is the most widely known enzymatic biosensor. The enzyme used to detect glucose is glucose oxidase (GOx). GOx catalyses glucose oxidation into gluconolactone and hydrogen peroxide [25]. Recently, Ha et al. [26] used an amperometry technique to check that there has been a substantial increase in the current response along with a quick increase in the glucose concentration.

### 2.1. Non-Enzymatic Glucose Sensor

**Figure 1:**
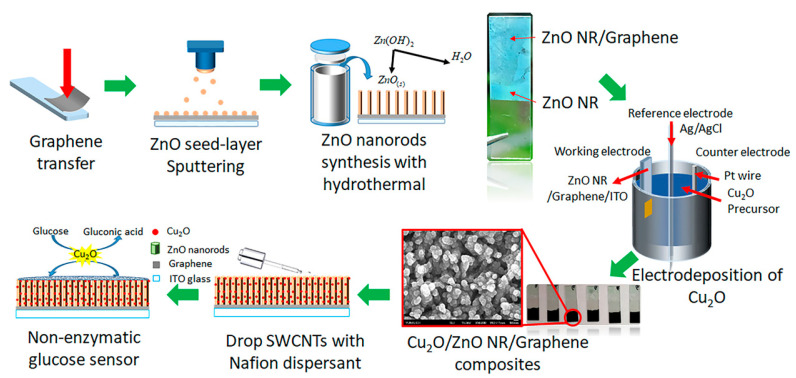
Synthesis steps of single-walled carbon nanotubes (SWCNTs)/Cu_2_O/ZnO NRs/graphene hybrid electrodes, including graphene transfer, ZnO NR synthesis with hydrothermal, Cu_2_O growth with electrodeposition, and drop SWCNTs with Nafion dispersant for nonenzymatic glucose sensors. Reproducedfrom Chen, H.C.; Su, W.R. and Yeh, Y.C., 2020. Functional channel of SWCNTs/Cu_2_O/ZnO NRs/graphene hybrid electrodes for highly sensitive nonenzymatic glucose sensors. ACS Appl. Mater. Interfaces, 12, pp. 32905–32914 [41].

**Figure 2:**
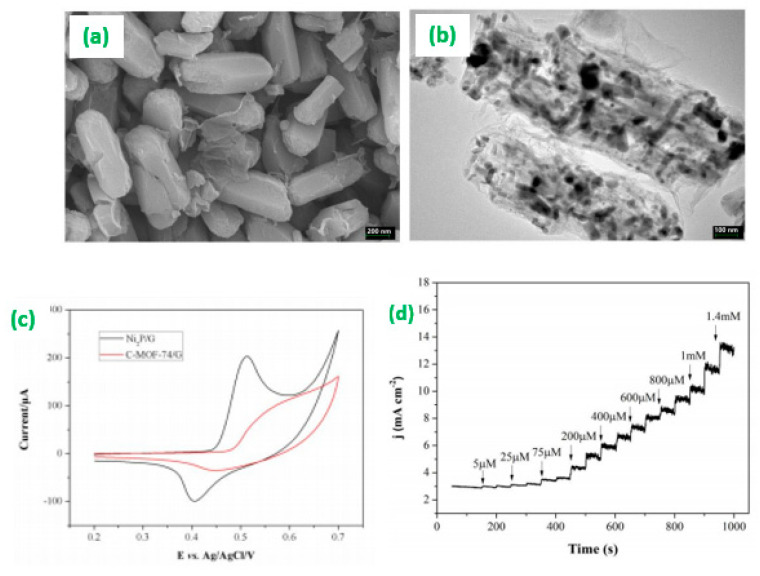
(**a**) SEM image and (**b**) TEM image of Ni_2_P/G, (**c**) CVs of Ni_2_P/G/GCE (black) and C-Ni-MOF-74/G/GCE (red), and (**d**) amperometric responses of Ni_2_P/G/GCE to successive additions of glucose (in 0.1 M NaOH solution with the potential of 0.5 V vs. Ag/AgCl electrode). Reproduced from Zhang, Y.; Xu, J.; Xia, J.; Zhang, F. and Wang, Z., 2018. MOF-Derived Porous Ni_2_P/Graphene Composites with Enhanced Electrochemical Properties for Sensitive Non enzymatic Glucose Sensing. ACS Appl. Mater. Interfaces, 10, pp. 39151–39160 [42].

Glucose is an extremely imperative nutrient that is used in main catabolic pathways of humanity. Worldwide, diabetes mellitus (DM) is one of the major diseases shortening human lifespan. DM is a collection of metabolic disorder characterized by hyperglycemia owing to insulin production defects and/or insulin action. As per the International Diabetes Federation (IDF), the global spread of DM evaluated patients would increase to 642 million by 2040, based on 10.4% of the global population [27]. Monitoring the blood glucose levels is the most important aspect. A high level of glucose, called hyperglycemia, ranges between 5.6 and 7 mM [28]. On other hand, the lower blood glucose level is 3.9 mM, which leads to a potentially deadly stage referred hypoglycemia [29]. Due to abnormal glucose levels, heart disease, renal failure, blindness, kidney failure, and stroke have been reported [30,31]. Consequently, it is essential to establish a fast, reliable, accurate and responsive blood sugar monitoring technique [32]. Especially, the electrochemical determination technique gives a rapid and highly cheap pathway to detect the glucose. The electrochemical detection of glucose sensor is developed on the glucose oxidase (GOx) enzyme. Though, some problems exhibited on the enzymatic glucose sensor owing to the inadequate stability, denaturation of enzymes, thermal and chemical instability and environmental factors like moisture, pH and temperature [33,34]. Thus, a simple and highly sensitive glucose sensor is required to detect glucose effectively. Considering the abovementioned difficulties, numerous researches have been reported on the non-enzymatic electrochemical glucose sensor. For instance, Chen et al. discussed a facile synthesis and material characterizations of NdNiO_3_ nanoparticles for a non-enzymatic glucose sensor application. The NdNiO_3_ nanoparticles prepared by facile synthesis display unique properties with excellent electrochemical efficiency and have a low detection limit of up to 0.3 mM, with an ultra-high sensitivity of 1105.1 mAmM^−1^cm^−2^. They were commercially suitable for real-time use in a non-enzymatic glucose sensor [35]. LaTiO_3_-Ag_0.2_ perovskite material was synthesized by exploiting particulate sol–gel process, and the morphological studies revealed that Ag adheres on the LaTiO_3_ surface. The electrochemical properties of LaTiO_3_-Ag_0.2_ was examined by CV, depicting an important glucose oxidation at +0.70 V vs. Ag/AgCl under NaOH electrolyte, this demonstrate effective formation of LaTiO_3_-Ag_0.2_. The as-fabricated glucose sensor provided a LOD, sensitivity and linearity of 0.21 µM, 784.14 µAmM^−1^cm^−2^, and 0.0025 to 4 mM, respectively, owing to enhanced actual surface area. Moreover, it displayed good selectivity and exhibited recovery ranges 101.3% to 102.6% in human blood serum [36]. Zheng et al. synthesized LaNi_0.6_Co_0.4_O_3_ perovskite using a sol–gel technique, and the morphological analysis revealed that the as-synthesized LaNi_0.6_Co_0.4_O_3_ possessed few fine granules and other large particles. The CV responses of the constructed LaNi_0.6_Co_0.4_O_3_/CPE displayed a noticeable glucose oxidation at 0.55 V vs. SCE, a sensitivity of 643 µAmM^−1^cm^−2^, a detection ranges between 0.05 and 200 µM and a low LOD of 8 nM owing to the many crystalline disorders in LaNi_0.6_Co_0.4_O_3_ [37]. Wang et al. developed perovskite material LaNi_0.5_Ti_0.5_O_3_ as an electrochemical probe for non-enzymatic glucose detection. The Ni (III) from LaNi_0.5_Ti_0.5_O_3_ produced under alkaline medium can oxidize the glucose and exhibited fast amperometric performances. It also demonstrated excellent selectivity and the recovery range of 95.6% to 101.9%, respectively [38]. La_0.6_Sr_0.4_CoO_3-δ_ perovskite was prepared by a sol-gel process using EDTA-citrate complex. The as-fabricated La_0.6_Sr_0.4_CoO_3-δ_+rGO/GCE sensor was applied for glucose sensing, which provided a quick amperometric response at 0.6 V vs. Ag/AgCl under alkaline medium, linearity, sensitivity, detection limit of 2 µM to 3.35 mM, 330 µAmM^−1^cm^−2^ and 63 nM, respectively. La_0.6_Sr_0.4_CoO_3-δ_+rGO demonstrated high electrocatalytic activity towards glucose due to an effective synergetic reaction mediated by Co redox coupled with oxygen vacancy [39]. Pr_1.2_Ba_0.08_Ni_0.95_Zn_0.05_O_4+δ_ perovskite, a form of A_2_BO_4+δ_, was first tested for non-enzymatic glucose detection. In terms of dynamic range (1.5–7000 µM) and detection limit (0.5 µM), the analytical performance of this glucose sensor is good compared to the previously published ABO_3_ perovskite modified electrodes. Its application of human serum indicates that there is no intervention in the detection of glucose [40]. Chen et al. reported four step preparations of single-walled carbon nanotubes (SWCNTs)/Cu_2_O/ZnO/graphene electrodes for non-enzymatic glucose detection, as shown in Figure 1. The fabricated hybrid electrodes delivered the significant improvement in sensitivity from 11.2 µAmM^−1^ cm^−2^ to 289.8 µAmM^−1^cm^−2^ and a linear detection range from 600 µM to 11.1 mM [41]. The synthesis of 3D Ni_2_P/G composite from the Ni-MOF-74/G composites using the phosphorization method was reported by Zhang et al. After phosphorization, the 3D Ni_2_P/G composite has a slight contraction and some rough surface when compared to the Ni-MOF-74/G composites. The result is that Ni_2_P nanoparticles are formed and organic ligands are partly pyrolysed (Figure 2a). The formation of Ni_2_P nanoparticles that are homogeneously dispersed on the 3D graphene is displayed in Figure 2b. The electrochemical performance of the prepared composites was investigated using cyclic voltammetry. The result shows that it has higher electrocatalytic activity due to a strong synergistic effect between Ni_2_P nanoparticles and graphene in 3D Ni_2_P composite (Figure 2c). The amperometric analysis showed an increased sensitivity of 7234 µA mM^−1^cm^−2^, wide detection range of 5 µM to 1.4 mM with a detection limit of 0.44 µM (Figure 2d) [42]. PtNi alloy NPs anchored graphene nanocomposites were synthesized by a facile one-pot ultrasonication aided electrochemical technique and showed significantly increased response current toward glucose due to bifunctional effects and electronic effects in PtNi/ERGO nanocomposites. The sensor accounts linearly for glucose up to 35 mM, with a limit of detection of 0.01 mM and a sensitivity of 20.42 µA mM^−1^cm^−2^ [43]. An innovative Co (OH)_2_ NRs were deposited over a 3-dimensional (3DG) graphene network through chemical bath deposition. The Co (NH_3_)_4_^2+^ can easily adsorb on the surface of the rippled and wrinkled 3DG by electrostatic force. Afterwards, the unstable Co (NH_3_)_4_^2+^ turned to Co (OH)_2_ over 3DG surface. Simple and rapid electron-transfer kinetics toward glucose oxidation were provoked at the Co (OH)_2_/3DG composites beneath alkaline medium because of its excellent conductivity and high electroactive surface area, as indicated by its high sensitivity. The fabricated Co(OH)_2_/3DG/GCE unveiled considerable act at an applied potential of 0.6 V in 1 M KOH towards glucose detection, which displayed excellent sensitivity, linear range and LOD of 3.69 mA mM^−1^ cm^−2^, 100 µM to 10 mM, and 16 nM, respectively [44].

Glucose is an extremely imperative nutrient that is used in main catabolic pathways of humanity. Worldwide, diabetes mellitus (DM) is one of the major diseases shortening human lifespan. DM is a collection of metabolic disorder characterized by hyperglycemia owing to insulin production defects and/or insulin action. As per the International Diabetes Federation (IDF), the global spread of DM evaluated patients would increase to 642 million by 2040, based on 10.4% of the global population [27]. Monitoring the blood glucose levels is the most important aspect. A high level of glucose, called hyperglycemia, ranges between 5.6 and 7 mM [28]. On other hand, the lower blood glucose level is 3.9 mM, which leads to a potentially deadly stage referred hypoglycemia [29]. Due to abnormal glucose levels, heart disease, renal failure, blindness, kidney failure, and stroke have been reported [30,31]. Consequently, it is essential to establish a fast, reliable, accurate and responsive blood sugar monitoring technique [32]. Especially, the electrochemical determination technique gives a rapid and highly cheap pathway to detect the glucose. The electrochemical detection of glucose sensor is developed on the glucose oxidase (GOx) enzyme. Though, some problems exhibited on the enzymatic glucose sensor owing to the inadequate stability, denaturation of enzymes, thermal and chemical instability and environmental factors like moisture, pH and temperature [33,34]. Thus, a simple and highly sensitive glucose sensor is required to detect glucose effectively. Considering the abovementioned difficulties, numerous researches have been reported on the non-enzymatic electrochemical glucose sensor. For instance, Chen et al. discussed a facile synthesis and material characterizations of NdNiO_3_ nanoparticles for a non-enzymatic glucose sensor application. The NdNiO_3_ nanoparticles prepared by facile synthesis display unique properties with excellent electrochemical efficiency and have a low detection limit of up to 0.3 mM, with an ultra-high sensitivity of 1105.1 mAmM^−1^cm^−2^. They were commercially suitable for real-time use in a non-enzymatic glucose sensor [35]. LaTiO_3_-Ag_0.2_ perovskite material was synthesized by exploiting particulate sol–gel process, and the morphological studies revealed that Ag adheres on the LaTiO_3_ surface. The electrochemical properties of LaTiO_3_-Ag_0.2_ was examined by CV, depicting an important glucose oxidation at +0.70 V vs. Ag/AgCl under NaOH electrolyte, this demonstrate effective formation of LaTiO_3_-Ag_0.2_. The as-fabricated glucose sensor provided a LOD, sensitivity and linearity of 0.21 µM, 784.14 µAmM^−1^cm^−2^, and 0.0025 to 4 mM, respectively, owing to enhanced actual surface area. Moreover, it displayed good selectivity and exhibited recovery ranges 101.3% to 102.6% in human blood serum [36]. Zheng et al. synthesized LaNi_0.6_Co_0.4_O_3_ perovskite using a sol–gel technique, and the morphological analysis revealed that the as-synthesized LaNi_0.6_Co_0.4_O_3_ possessed few fine granules and other large particles. The CV responses of the constructed LaNi_0.6_Co_0.4_O_3_/CPE displayed a noticeable glucose oxidation at 0.55 V vs. SCE, a sensitivity of 643 µAmM^−1^cm^−2^, a detection ranges between 0.05 and 200 µM and a low LOD of 8 nM owing to the many crystalline disorders in LaNi_0.6_Co_0.4_O_3_ [37]. Wang et al. developed perovskite material LaNi_0.5_Ti_0.5_O_3_ as an electrochemical probe for non-enzymatic glucose detection. The Ni (III) from LaNi_0.5_Ti_0.5_O_3_ produced under alkaline medium can oxidize the glucose and exhibited fast amperometric performances. It also demonstrated excellent selectivity and the recovery range of 95.6% to 101.9%, respectively [38]. La_0.6_Sr_0.4_CoO_3-δ_ perovskite was prepared by a sol-gel process using EDTA-citrate complex. The as-fabricated La_0.6_Sr_0.4_CoO_3-δ_+rGO/GCE sensor was applied for glucose sensing, which provided a quick amperometric response at 0.6 V vs. Ag/AgCl under alkaline medium, linearity, sensitivity, detection limit of 2 µM to 3.35 mM, 330 µAmM^−1^cm^−2^ and 63 nM, respectively. La_0.6_Sr_0.4_CoO_3-δ_+rGO demonstrated high electrocatalytic activity towards glucose due to an effective synergetic reaction mediated by Co redox coupled with oxygen vacancy [39]. Pr_1.2_Ba_0.08_Ni_0.95_Zn_0.05_O_4+δ_ perovskite, a form of A_2_BO_4+δ_, was first tested for non-enzymatic glucose detection. In terms of dynamic range (1.5 µM–7000 µM) and detection limit (0.5 µM), the analytical performance of this glucose sensor is good compared to the previously published ABO_3_ perovskite modified electrodes. Its application of human serum indicates that there is no intervention in the detection of glucose [40]. Chen et al. reported four step preparations of single-walled carbon nanotubes (SWCNTs)/Cu_2_O/ZnO/graphene electrodes for non-enzymatic glucose detection, as shown in Figure 1. The fabricated hybrid electrodes delivered the significant improvement in sensitivity from 11.2 µAmM^−1^ cm^−2^ to 289.8 µAmM^−1^cm^−2^ and a linear detection range from 600 µM to 11.1 mM [41]. The synthesis of 3D Ni_2_P/G composite from the Ni-MOF-74/G composites using the phosphorization method was reported by Zhang et al. After phosphorization, the 3D Ni_2_P/G composite has a slight contraction and some rough surface when compared to the Ni-MOF-74/G composites. The result is that Ni_2_P nanoparticles are formed and organic ligands are partly pyrolysed (Figure 2a). The formation of Ni_2_P nanoparticles that are homogeneously dispersed on the 3D graphene is displayed in Figure 2b. The electrochemical performance of the prepared composites was investigated using cyclic voltammetry. The result shows that it has higher electrocatalytic activity due to a strong synergistic effect between Ni_2_P nanoparticles and graphene in 3D Ni_2_P composite (Figure 2c). The amperometric analysis showed an increased sensitivity of 7234 µA mM^−1^cm^−2^, wide detection range of 5 µM to 1.4 mM with a detection limit of 0.44 µM (Figure 2d) [42]. PtNi alloy NPs anchored graphene nanocomposites were synthesized by a facile one-pot ultrasonication aided electrochemical technique and showed significantly increased response current toward glucose due to bifunctional effects and electronic effects in PtNi/ERGO nanocomposites. The sensor accounts linearly for glucose up to 35 mM, with a limit of detection of 0.01 mM and a sensitivity of 20.42 µA mM^−1^cm^−2^ [43]. An innovative Co (OH)_2_ NRs were deposited over a 3-dimensional (3DG) graphene network through chemical bath deposition. The Co (NH_3_)_4_^2+^ can easily adsorb on the surface of the rippled and wrinkled 3DG by electrostatic force. Afterwards, the unstable Co (NH_3_)_4_^2+^ turned to Co (OH)_2_ over 3DG surface. Simple and rapid electron-transfer kinetics toward glucose oxidation were provoked at the Co (OH)_2_/3DG composites beneath alkaline medium because of its excellent conductivity and high electroactive surface area, as indicated by its high sensitivity. The fabricated Co(OH)_2_/3DG/GCE unveiled considerable act at an applied potential of 0.6 V in 1 M KOH towards glucose detection, which displayed excellent sensitivity, linear range and LOD of 3.69 mA mM^−1^ cm^−2^, 100 µM to 10 mM, and 16 nM, respectively [44].

### 2.2. Electrochemically Sensing of Hydrazine and Dopamine

**Figure 3:**
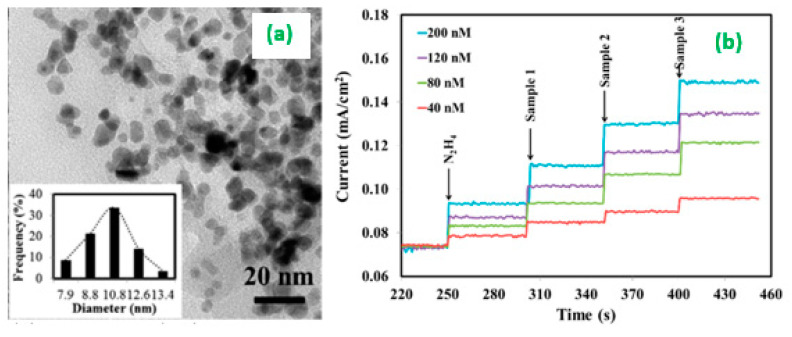
(**a**) TEM images of PdNPs/rGO nanocompostie and (**b**) chronoamperomograms of the PdNPs/rGO RDE at a rotation rate of 6000 rpm with consecutive sample injections. Reproducedfrom Krittayavathananon, A.; Srimuk, P.; Luanwuthi, S. and Sawangphruk, M, 2014. Palladium nanoparticles decorated on reduced graphene oxide rotating disk electrodes toward ultrasensitive hydrazine detection: effects of particle size and hydrodynamic diffusion, Anal. Chem., 86, pp. 12272–12278 [52].

Hydrazine is broadly used in numerous fields, such as pharmaceutical, agricultural, and chemical applications as a chemical corrosion inhibitor, very reactive base, and a robust reducing agent [45,46]. It has been employed in rocket propellant, corrosion inhibitors, pesticides, insecticides, antioxidants, emulsifiers, explosives, oxygen scavenger, and herbicides, and so on. However, the World Health Organization (WHO) has stated that it is a potential carcinogen with a low TLV of 10 ppb capable of provoking carcinogenesis and mutagenesis and severely affecting the liver, kidneys, central nervous system, and lungs. It is also very toxic and can simply be absorbed by the oral respiratory tract of the skin [47,48]. In this situation, electrochemical techniques have emerged as a potential technique for hydrazine detection. Ali et al. constructed the rhombohedral LaCoO_3_ perovskite using the microwave-assisted citrate technique. Prepared LaCoO_3_ perovskite showed a well-arranged scaffold of bone-shaped grains with an average particle size of 140.4 nm. The electrochemical properties of LaCoO_3_ for hydrazine were investigated by CV, which observed hydrazine oxidation under alkaline medium by the presence of Co ions at the perovskite B-site. GC/LaCoO_3_ delivered high sensitivity; a linear range; and LOD of 5.61 mA mM^−1^ cm^−2^, 0.1 mM to 60 mM, and 0.15 mM, respectively. The high electrocatalytic activity of the resulting composite is owing to oxygen vacancy as defect and the presence of OH^−^ groups, which made facilitated the absorption of oxygen on the LaCoO_3_ surface [49]. Ali et al. acquired a SrPdO_3_ synthesized by the sol-combustion citrate technique and showed the orthorhombic morphology of the nano phase. SrPdO_3_ perovskite toward hydrazine oxidation electrocatalytic behavior was investigated using CV in neutral medium and showed a good sensitivity of 2.1 mA μM and a calculated LOD for hydrazine of 83 pM [50]. The rGO/CuO nanocomposites were synthesized by hydrothermal technique and exploited as effective catalysts for electrocatalytic activity to detect hydrazine. Morphological images revealed that the acquisitions of CuO NRs were acquired with average diameter and length of 18 nm and 130 nm, which are homogeneously dispersed and effectively anchored over the rGO network. Amperometric analysis at +0.40 V vs. Ag/AgCl under neutral conditions, where the composite exhibited a sensitivity of 3.87 μA μM^−1^ cm^−2^, a linear range of 0.1 to 400 M and a detection limit of 9.8 nM due to the highest density of Cu^2+^ sites in (200) planes, which is useful for the hydrazine oxidation and enhanced electrocatalytic activity attained through robust synergistic interactions of the two components [51]. Insitu polyol technique was used to prepare palladium nanoparticles/reduced graphene oxide (PdNPs/rGO) composites. The prepared nanocomposites, PdNPs effectively dispersed over the rGO sheets. As-constructed PdNPs/rGO rotating disk electrodes (RDEs) were examined for hydrazine detection. PdNPs/rGO RDE delivered an excellent low detection limit of 7 nM at 6000 rpm and provided a linear range of 0.1 to 1000 μM at 2000 rpm owing to the highest active surface area-to-volume ratio (Figure 3) [52].

Dopamine belongs to a class of catechol amines and is the most significant neurotransmitter in the mammalian central nervous systems and peripheral nervous systems, managing a diversity of neuronal functions such as stress, movement, attention, behavior, learning, and memory [53]. Insufficient levels of dopamine in the blood cause Parkinson’s disease and higher levels of dopamine can cause high pressure, euphoria, and schizophrenia. In this context, the electrochemical technique can be significant for dopamine detection [54,55]. Berry-like NaNbO_3_ perovskite nanomaterials have been prepared using solvothermal method. Morphological studies of as-synthesized nanomaterials have shown that these berry-like NaNbO_3_ perovskite have an average particle size of ~96 nM to ~296 nM. The above-mentioned nanomaterials proved to be effective sensors towards detection of dopamine at ~0.12 V due to the enhanced specific surface area and increased electron transport. It exhibited a wide linear range between 10 nM to 500 mM, a high sensitivity of 99 nA nM^−1^ cm^−2^ and a LOD of 6.8 nM for dopamine sensing [56]. Atta et al. reported CpE/SrPdO_3_ electrode for dopamine electrochemical sensing and assessed its electrochemical behavior under neutral medium. The CpE/SrPdO_3_ electrode exhibited a distinctive long-term stability and LOD for dopamine detection. It showed two linear ranges of 7 to 70 μM and 90 to 160 μM with low detection limits of 9.3 nM and 25 nM [57]. β-NaFeO_2_ perovskite was prepared by calcination technique. As-synthesized β-NaFeO_2_ materials were homogenously distributed with pebble like morphology and particles ranging in size from ~90 nm to ~250 nm. The constructed β-NaFeO_2_/GCE exhibited outstanding selectivity towards dopamine against other interference species and displayed an oxidation potential at +0.2 V and a LOD of 2.12 nM for DA. The real sample analysis from human blood serum showed the recovery range from of 98.4% to 103.27% [58]. Lanthanum ferrite (LaFeO_3_) was achieved using a solid-state method. The constructed sensor has two distinctive ranges from 10 µM to 100 µM and 120 µM to 180 µM and a LOD of 10 nM. The excellent performances of the dopamine sensor were due to the enlarged surface area and more electro active sites in LaFeO_3_ [59]. Thirumalirajan et al. reported simple preparation of LaFeO_3_ microspheres through a single-step chemical approach. The morphological studies depicted that the LaFeO_3_ microspheres possessed particles size between 0.5 and 1.5 µm. Constructed LaFeO_3_/GCE has unveiled a significant performance towards the detection of dopamine which has a high sensitivity at a low detection limit of 59 nM and wide linear range from 20 nM to 1.6 μM [60]. A new route to construct electrochemically reduced α-Fe_2_O_3_@erGO nanocomposite as the working electrode material to the electrochemical determination of dopamine has been reported. The prepared α-Fe_2_O_3_@erGo nanocomposite was used for dopamine detection, showing a linear dynamic range from 0.25 to 100 μM in response to DA with a LOD of 0.024 μM. This sensor exhibited high performances due to positive dopamine electrostatically interacting with negatively charged α-Fe_2_O_3_@erGo surface via π–π stacking which supports increased electron transport during an electrochemical catalytic reaction [61]. The rGO/ZIF-8 composite was synthesized by a facile chemical reduction approach. The fabricated rGO/ZIF-8 depicted large sensitivity for the determination of DA. Moreover, a linear response range of 0.1 μM to 0.1 mM and 30 nM was observed [62]. Cheng et al. synthesized graphene-MoS_2_ nanocomposites utilizing a facile and effective technique. A dopamine electrochemical sensor was simply constructed by graphene-MoS_2_ GCE exhibited a linear range and a detection limit of 50 nM to 10 µM and 7.13 nM, respectively, under neutral condition due to the excellent electron transport capability of graphene-MoS_2_ [63].

### 2.3. Electrochemical Sensing of Hydrogen Peroxide

**Figure 4:**
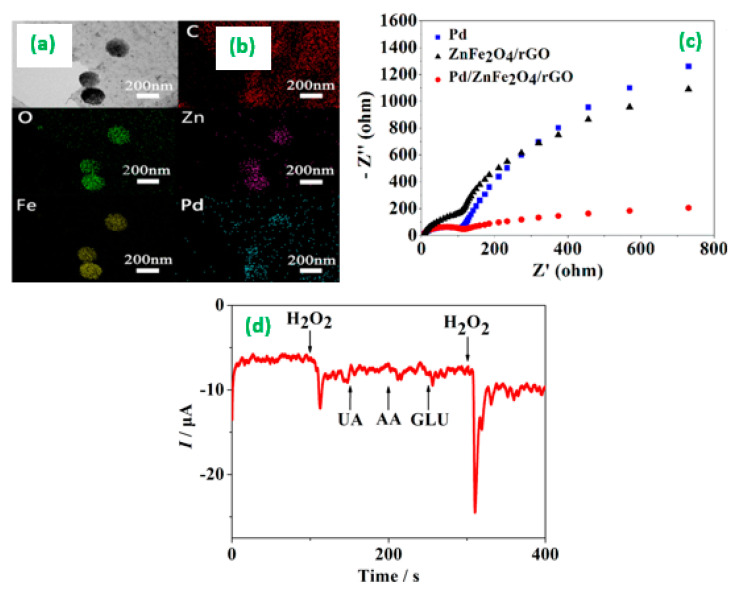
(**a**) TEM image of Pd/ZnFe_2_O_4_/rGO; (**b**) and corresponding elemental mapping images of C, O, Zn, and Fe; (**c**) Nyquist plots of different electrodes in 5.0 mM [Fe(CN)_6_]^4^^−/3^^−^ solution containing 0.2 M KCl and Pd; and (**d**) Amperometric response to successive additions of 0.1 mM H_2_O_2_, 0.1 mM UA, 0.1 mM AA, 0.1 mM GLU, and a second 0.1 mM H_2_O_2_ of Pd/ZnFe_2_O_4_/rGO at −0.2 V in 0.1 M PBS. Reproducedfrom Ning, L.; Liu, Y.; Ma, J.; Fan, X.; Zhang, G.; Zhang, F.; Peng, W. and Li, Y., 2017. Synthesis of palladium ZnFe_2_O_4_ functionalized reduced graphene oxide nanocomposite as H_2_O_2_ detector, Ind. Eng. Chem. Res., 56, 15, pp. 4327–4333 [70].

**Figure 5:**
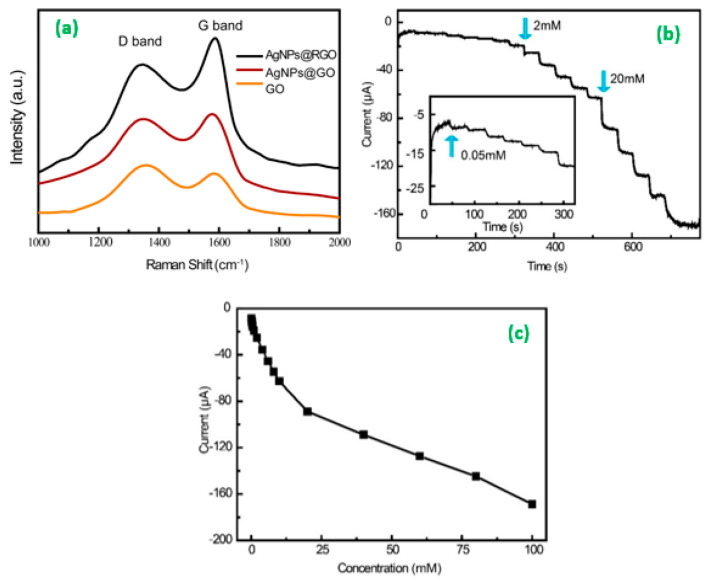
(**a**) Raman spectra of GO, AgNPs@GO. (**b**) Time–current response of the AgNPs@RGO/ITO electrode with a successive injection of H_2_O_2_ at the applied potential of −0.1 V. (**c**) The corresponding calibration plot for H_2_O_2_ detection. Reproduced from Zhu, L.; Gao, Y.Y.; Han, B.; Liu, S.; Fu, X.Y.; Dong, H. and Zhang, Y.L., 2019. Programmable laser patterning of Ag nanoparticles and reduced graphene oxide hybrid electrode for non enzymatic hydrogen peroxide detection, ACS App. Nano. Mater., 2, 12, pp. 7989–7996 [71].

In general, the moderate concentration of H_2_O_2_ to maintain the biological system while increasing the abnormal concentration of H_2_O_2_ concentration tends to affect Parkinson diseases [64]. Simple, fast, and accurate measurement of hydrogen peroxide (H_2_O_2_) holds promising molecules of special interest in research and importance in various fields in the pharmaceutical, food industries, clinical diagnosis and environmental studies [65]. Various types of analytical techniques have been used to estimate H_2_O_2_ at low concentrations such as electrochemical [66], spectrophotometry [67] and fluorescence [68], respectively. Among them is an electrochemical technique that can be used as a best owing to its cost-effectiveness, simplicity, and high sensitivity [69]. New palladium synthesis supported ZnFe_2_O_4_ functionalized reduced graphene oxide (Pd/ZnFe_2_O_4_/rGO) composite by solvothermal method. As-prepared composites show nanospheres as porous structures tend to increase their contact between electrode and electrolyte solutions. Typical electrochemical impedance spectroscopic (EIS) analysis of Pd/ZnFe_2_O_4_/rGO composites, which showed the lowest charge-transfer resistance value (140.4 Ω), which may increase their conductivity. The environmentally friendly Pd/ZnFe_2_O_4_/rGO nanocomposite exhibited excellent electrocatalytic activity (sensitivity value is 621.64 mA mM^−1^ cm^−2^) for H_2_O_2_ detection (Figure 4) [70]. The unique morphology of AgNPs@RGO composite film electrodes is a key feature and a promising factor in the development of high sensitivity biosensors. Furthermore, the SP^3^-hybridized carbon defects of the internal layered graphite carbon and their layered dopamine characteristic bands (D band @ 1350 and G band at 1590 cm^−1^) have been successfully confirmed by the Raman spectrum. AgNPs@RGO composite film electrode demonstrated remarkable electrochemical performance for ultrasensitive H_2_O_2_ detection (Figure 5) [71]. Meanwhile, the novel two-dimensional (2D) and three-dimensional (3D) highly porous reduced copper oxide-based graphene oxide (3D Cu_2_O-GA and 2D Cu_2_O-rGO) composites have been synthesized using both cost-effective hydrothermal and filtration methods. The free-standing porous as composites gained a lot of interest due to the high electrochemical sensing properties of H_2_O_2_ detection [72]. Recently, the atom-thick PtNi nanowire, which is assembled on a reduced graphene oxide (PtNi NWs/rGO) electrode, has been widely used as a promising electrocatalytic material to significantly detect and boost the performance of the H_2_O_2_ sensor [73]. Luque et al. [74] reported that the catalytic behavior of the perovskite (La_0.66_Sr_0.33_MnO_3_-CPE) electrode was associated with the amperometric sensing of H_2_O_2_. Karuppiah et al. [75], have deliberate the electrocatalytic reduction of H_2_O_2_ sensing using the electroactive LaMnO_3_ based carbon black (CB) electrode catalyst by dry particle coating method. They have confirmed its superior electrochemical properties with good sensitivity and H_2_O_2_ detection limit. Baghayeri et al. [76] have constructed a novel H_2_O_2_ biosensor based on functionalized graphene oxide supported amine terminated poly(amidoamine) dendrimer modified palladium nanoparticle (GO-Fe_3_O_4_-PAMAM-Pd) composite electrode. The well-defined cathodic behavior of GO-Fe_3_O_4_-PAMAM-Pd shows good electrocatalytic activity towards the reduction of H_2_O_2_.

## 3. Gas Sensors

### 3.1. Hydrogen Sulfide Sensor (H_2_S)

Hydrogen sulfide (H_2_S) is a poisonous, pungent, and incredibly annoying chemical. The higher concentration for H2S subjection over one hour without severe repercussions was calculated to be nearly as 170 to 300 ppm. Organic products, renewable energy, thermal gas, gasoline, wastewater and sulfur deposits are the prime causes of H_2_S gas [77]. An extremely sensitive, selective, and efficient H_2_S sensor is of high importance for human welfare protection. For the last few decades, studies on harmful gas monitoring has generally focused on the use of gas sensors made of different usable materials such as carbon-based nanomaterials [78], metal oxide/metallic nanostructures [79], transition metal dichalcogenides (TMDs) [80], gallium nitride (GaN) [81], organic materials [82], solid electrolytes [83], zeolites [84], and others [85]. The advances in electrochemical sensors for H_2_S detections have been examined here with regards to specific sensor materials. Essential sensing factors such as ppm concentration, stability, reaction time, sensitivity and operating temperature of numerous sensor materials are summarized.

**Figure 6:**
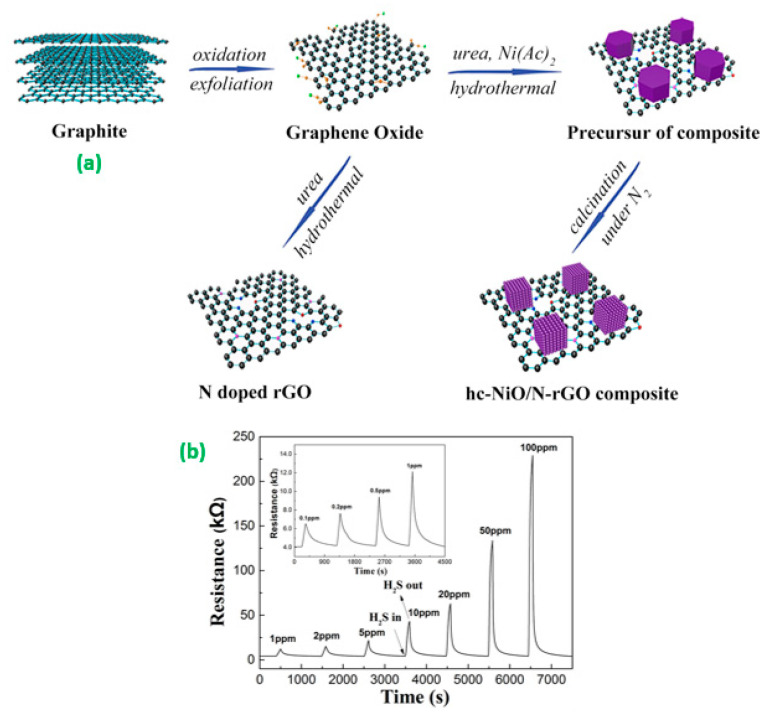
(**a**) Schematic preparation diagram of the hc-NiO/NrGO Composite Material and (**b**) response and recovery curve of the hc-NiO/N-rGO-based sensor toward 0.1–100 ppm of H_2_S at 92 °C. Reproduced from Yang, M.; Zhang, X.; Cheng, X.; Xu, Y.; Gao, S.; Zhao, H. and Huo, L., 2017.Hierarchical NiO Cube/Nitrogen-Doped Reduced Graphene Oxide Composite with Enhanced H_2_S Sensing Properties at Low Temperature, ACS Appl. Mater. Interfaces, 9, pp. 26293–26303 [91].

The carbon materials can be selected to improve the efficiency of the gas reaction due to their large specific surface areas and superior ionic conductivity. For example, Choi et al. [86] synthesized a SnO_2_/RGO composite nanofiber with a high sensitivity and selectivity at about 5 ppm H_2_S at 200 °C by means of an electrospinning method. For H_2_S chemiluminescence sensing, Jiang et al. [87] described a paper-like Fe_2_O_3_/graphene composite developed employing a supercritical CO_2_-assisted thermal process with a detection limit of 10 ppm at 130 °C. Bai et al. [88] synthesized a nanorod MoO_3_/graphene composite using microwave assisted procedure, and the optimum reaction to 40 parts per million of H_2_S at 110 °C was 59.7, that is considerably larger than the undoped composite. The composite Co_3_O_4_/single-walled carbon nanotube (SWCNT) was prepared by Moon et al. [89] and reported a lower response of 100 ppm of H_2_S (5.9), a relatively higher temperature (250 °C), and a better limit of detection (5 ppm). Xu et al. (2019) [90] synthesized a composite of Co_3_O_4_ hollow nanosphere/graphene with a size of about 15 nm that is equitably moored by cross-linking deposition on the graphene layers. Moreover, within the range of 0.1 to 100 ppm the response had the outstanding linear correlation to the H_2_S content. An innovative hierarchical composite of NiO cube (hc-NiO)/nitrogen-doped reduced graphene oxide (N-rGO) is synthesized by a facile hydrothermal route and a post-calcination procedure without the addition of templates and surfactants [91].The fabricated composite sensor has a stronger sensitivity to 0.1−100 ppm of H_2_S at a lower temperatures of 92 °C, and a strong linear response at different H_2_S concentrations, as seen in Figure 6.Efficient sensing of H_2_S can be due to a peculiar high porosity nature and many effective adsorption surface sites. The hybrid composite uses both materials to create high performance material H_2_S ultra-sensitivity materials.

**Figure 7:**
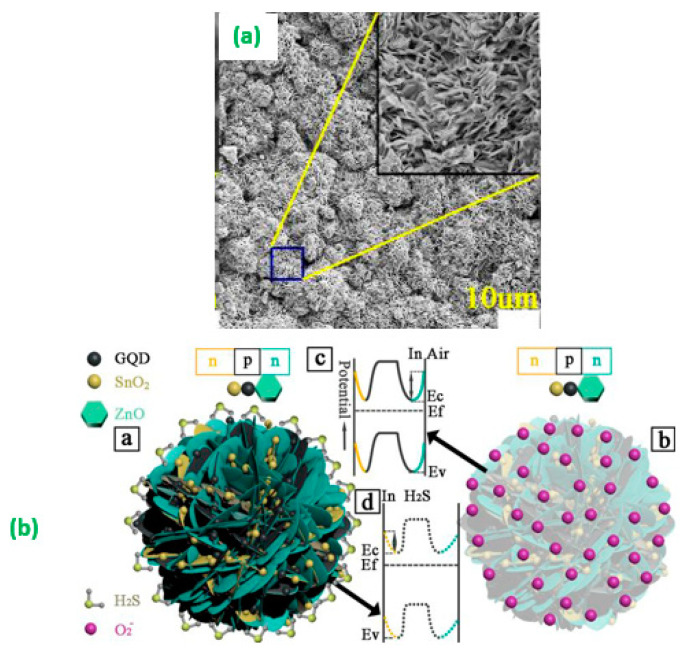
(**a**) SEM images of GQD-SnO_2_/ZnO nanosheet with 6 h psHT and (**b**) schematic diagram of band configuration at the interface of GQD-SnO_2_/ZnO nanostructure in different atmospheres. Reproduced from Shao, S.; Chen, X.; Chen, Y.; Zhang, L.; Kim, H.W. and Kim, S.S., 2020. ZnO Nanosheets Modified with Graphene Quantum Dots and SnO_2_ Quantum Nanoparticles for Room-TemperatureH_2_S Sensing, ACS Appl. Nano Mater., pp. 1–11 [93].

Sridhar et al. [92] used electrochemical impedance spectroscopy (EIS) to evaluate the sensor’s H_2_S gas responses formed by reduced graphene oxide incorporated nano-zinc oxide (n-ZnO) composites. The fabricated composites have been effective in detecting H_2_S at levels ranges from 2 to 100 ppm at 90 °C. A study by Shao et al. [93] devised a self-assembly technique to create functionalized porous and hierarchical SnO_2_ quantum nanoparticles. The GQD-modified hierarchical nanostructure displayed a significantly high response (S = 15.9 for 0.1 ppm H_2_S), swift period of response (14/13s), and strong H_2_S efficacy against other interrupting gases. It also has a large capacity for noninvasive exhaled treatment (Figure 7). The effect of Fe-dopants on the gas-sensing processes of the CCTO sensor has been described on the basis of the electronic and catalytic impacts of the substitution of p-type dopants [94]. Jang et al. [95] have researched the fabrication of poly(4-styrenesulfonic acid)-doped polyaniline/graphene (PSS-doped PANI/graphene) nanocomposites and their utilization as sensing components for the detection of hydrogen sulfide (H_2_S) showing that 168.4 S cm^−^^1^ conductivities and 13.1 Ω surface resistances have been obtained, demonstrating the suitability of solution-processed electrodes for H_2_S sensor networks. Lee et al. [96] have prepared a NiO ÿanodiscs hybrid nanocomposite via a facile hydrothermal procedure. At room temperatures, the hybrid nanocomposite-based sensor has enhanced sensing efficiency and is between ~2 and 3 times greater than the sensor response of other composites. In another investigation, Zhou et al. [97] prepared the rGO/Cu_2_O sensor, which displayed a 20% reaction to 1 ppm H_2_S, optimum recovery, excellent reproducibility, enduring stability, and selectivity. These encouraging results opened the way for the continued advancement for the identification of H_2_S sub-ppm and the potential implementation of monitoring of food freshness and extremely low emissions. Liu et al. [98] prepared SnO_2_ quantum wire/reduced graphene oxide nanocomposites using a single-step hydrothermal method. The significance of rGO in improving the SnQ_2_ QW gas sensors for H_2_S sensing has been discussed in detail. Furthermore, this analysis provides a quantitative framework to understand the gas-sensing process that is extensively needed for the production of gas sensors with lower energy consumption. Van Hieu et al. [99] studied the influence of calcination temperature, reaction solution content and RGO content on morphologies, properties, and gas sensing features of α-Fe_2_O_3_ NFs and RGO-loaded NFs fabricated using an on-chip electrospinning method. They found that the high sensitivity of the sensors was due to the inclusion of nanograins and reduced graphene oxide resulting in a large surface-to-volume ratio and the existence of possible barriers between nanograins at homojunctions and at heterojunctions between RGO and α-Fe_2_O_3_. The gas sensing experiments with the 0.5 wt% RGO-loaded CuO NFs sensor indicated that it has the highest response to H_2_S gas which could be used to design inexpensive and high efficiency H_2_S gas sensors [100]. Van Hoang et al. [101] recently synthesized sensors based on RGO-loaded ZFO NFs using an electrospinning method. The findings showed that the sensor calcined at 600 °C had the best response to H_2_S at specified gas concentration level and operational temperature ranges. Shi and coauthors [102] synthesized composites of rGO/h-WO_3_ nanosheets via a facile synthesis. The composite sensors displayed ameliorated H_2_S sensing performance compared to the undoped sensor. It also possess remarkable gas sensing characteristics, including good stability, slow reaction time, low detection range (10 ppb), wide linear detection range and selectivity, which could be due to hetero-junction formation, good acceptance/transportation of rGO electrons and efficient 3D hybrid nanostructure gas transmission channels. A high-performance sensor using SnO_2_-rGO nanocomposite as the sensing material for H_2_S and SOF_2_ sensing was introduced by Yang et al. [103]. The high-performance gas sensors were able to be deployed for online GIS tracking. Lee and coworkers [104] reported a selective highly sensitive hydrogen sulfide gas sensor based on graphene decorated with silver nanoparticles and charged impurities fabricated using a simple wet chemical method. Their research has shown that H_2_S adsorption and dissociation sites on the graphene surface have been induced by doping, enabling them to regulate the H_2_S content in real time and obtain an appropriate reaction at room temperature. Chaudhari et al. [105] prepared a tetragonal structured double perovskite Sr_2_Fe_0.6_Ni_0.4_MoO_6_ using a sol–gel citrate method. The inclusion of 0.5 wt% Pd increases the response of the gas sensor and decreased temperature coefficient of the H_2_S gas. Sol–gel Ni-doped CCTO films have extremely appealing sensing characteristics, including high H_2_S selectivity, appropriate H_2_S sub-ppm detection sensitivity and good reproducibility [106]. Chumakova et al. [107] synthesized a LaCoO_3_ nanocrystalline with a size of particles of 60–70 nm and a specific surface area of 5–10 m^2^/g using the process of sol–gel method using ethylenediamine as a coordination ligand which also demonstrated a higher sensitivity to H_2_S. The impact of the MoO_3_ content and the graphene oxide suspension concentration on the sensor response was probed by MalekAlaie et al. [108]. It has been observed that the prepared sensor has the greatest sensitivity. Decorated reduced graphene oxide chemiresistors deliver significant benefits including extraordinary mass production capability due to its flexibility, reasonable efficiency, and remarkable reactivity.

### 3.2. Ammonia (NH_3_) Sensor

Affordable, stable, and fast response sensors are deemed vital for numerous tracking options to reduce future human health and survival risks [109,110]. Ammonia (NH_3_) is a hazardous and combustible environmental pollutant that creates a continuous peril to commercial and domestic safety if its threshold level reaches 25 ppm in not less than 8 h [111]. NH_3_leads to environmental pollution from manmade and organic compounds sources, like automobile emissions [112], industrial activity [113], and soil manure [114]. Important attempts have therefore been made to produce ammonia-sensing materials that are minimal in scale, compact, and exceptionally sensitive in atmospheric and health applications at room temperature. Recently, flexible miniaturized gas sensors have earned considerable prominence over rigid sensors due to their cost effectiveness, corrosion resistance, and their capability to be assimilated into compact and lightweight electronic equipment [115]. Researchers noted that gas sensors embedded with such instruments are necessary to work at room temperature in order to maintain low-energy demand and exemplary features in terms of stability. In order to further extend the use of ammonia gas sensors, the sensors must preferably work at low temperatures; be lightweight, accessible, and fabric based; and they must be capable of working under high compressive force or large shape-deformation [116].

**Figure 8:**
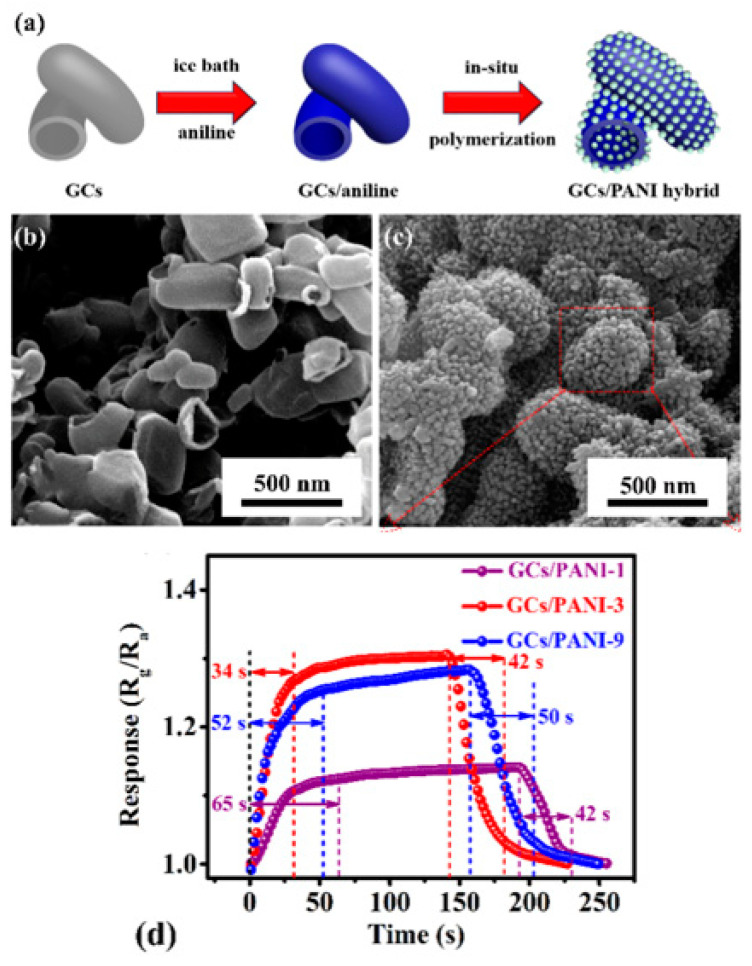
(**a**) Schematic illustrating the fabrication of the GCs/PANI hybrid composites. Scanning electron microscopy (SEM) images of the (**b**) pure GCs and (**c**) GCs/PANI-3 hybrid. (**d**) Dynamic response–recovery curves of the GCs/PANI-1-, GCs/PANI-3-, and GCs/PANI-9-based sensors toward 10 ppm NH_3_. Reproduced from Wang, H.; Nie, S.; Li, H.; Ali, R.; Fu, J.; Xiong, H.; Li, J.; Wu, Z.; Lau, W.M.; Mahmood, N.; Jia, R.; Liu, Y. and Jian, X., 2019.3D Hollow Quasi-Graphite Capsules/Polyaniline Hybrid with a High Performance for Room-Temperature Ammonia Gas Sensors, ACS Sens., 4, pp. 2343–2350 [120].

Two-dimensional (2D) nanocomposite materials, namely, graphene-based materials as electrochemical sensors have continued to receive a lot of interest due to their numerous rewards such as high surface area and easy structural regulation [117]. Pani, Pani/NiO, Pani/GN, and Pani@GN/NiO were systematically investigated by Mohammad et al. [118]. It was observed that under isothermal and cyclic ageing conditions, the nanocomposite illustrated the better thermal properties in terms of dc electrical conductivity retention which could be due to the electrostatic interaction between NiO, GN, and Pani. Jiao et al. [119] developed a reactive graphene-based ink sensor for the detection of NO_2_ and NH_3_ gas at room temperature. The sensors show reduced fragile nature and humidity levels, resulting in increased stability under different conditions. This ink represents a useful avenue for multi-gas sensors at ambient temperature, with extensive insights in the area of atmospheric control, industrial development, and clinical issue. Developing sensing components with novel morphologies and combinations is a major challenge in achieving high-performance gas sensor devices. At room temperature [120], a nanostructured GCs/PANI hybrid composite was grown and evinced an outstanding response to ammonia. It also displayed a strong reaction value of 1.30 and a recovery time of 34/42 s to 10 ppm of NH_3_ gas. As can be shown from Figure 8, the sensor demonstrated outstanding stability at room temperature. The extremely goodNH_3_-sensing performance can be due to the 3D hierarchical hybrid configuration and synergistic influence of PANI and GCs. 

**Figure 9:**
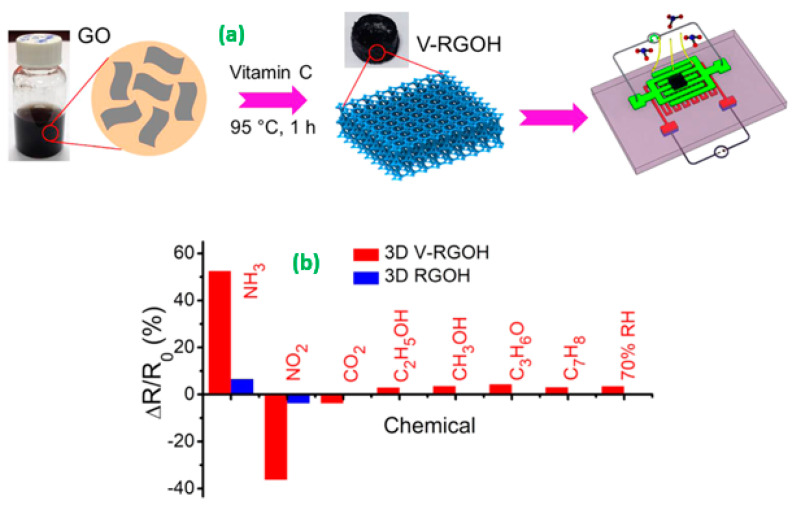
(**a**) Schematic depicting the green synthesis of 3D V-RGOH from GO with the aid of VC via self-assembly, for gas detection. Insets: photos of the GO aqueous dispersion (left) and 3D V-RGOH (middle). The chemiresistor and microheater were generated on the front and back sides of the Si/SiO_2_ substrate, respectively. (**b**) Responses of 3D V-RGOH and RGOH to 100 ppm NH_3_; 10 ppm NO_2_; 500 ppm CO_2_; saturated ethanol, methanol, acetone, and toluene vapors; and 70% RH. Reproduced from Wu, J.; Wei, Y.; Ding, H.; Wu, Z.; Yang, X.; Li, Z.; Huang, W.; Xie, X.; Tao, K. and Wang, X., 2020. Green Synthesis of 3D Chemically Functionalized Graphene Hydrogel for High-Performance NH_3_ and NO_2_ Detection at Room Temperature, ACS Appl. Mater. Interfaces, 12, pp. 20623–20632 [121].

Wu et al. [121] synthesized a VC-functionalized 3D RGOH over an environmentally friendly, simple, and mild self-assembly route with superior ammonia and nitrogen dioxide sensing output at room temperature. The prepared sensor showed enhanced linearity, good selectivity, a huge range of detection, and high resistance to moisture (Figure 9). This analysis represents a new opportunities to not only design and build high efficiency products for gas sensing through a natural, easy, and cost-beneficial technique but to also streamline RGO’s gas detecting properties by coupling chemical modifications with 3D structural control. MXene/rGO fibers [122] offer the highest mechanical strength, sustainability, and chemical response at room temperature for promising applications in lightweight and portable gas sensors. In another study, Tang et al. [123] developed an ammonia (NH_3_) sensor, in which the ultrathin polypyrrole (PPy) substrate is synthesized by electropolymerization on reduced graphene oxide. The sensor displays a steady NH_3_ response at normal temperature varies from 1 to 4 ppm, with a response of 6.1%/ppm, retrieval times of 1 and 5 min, respectively. The present sensor is promising as a smart sensor device for various implementations due to its uneconomic, good battery life and scalable output. The single-yarn NH_3_ detection system made of (GO/PAH)_1_ multilayer thin film [124] produced the highest reactivity, good LOD (1.5 ppm), maximal homogeneity, short response time (68 s), high versatility, high flexibility and high efficiency over a diverse set of NH_3_ gas concentrations, 5–100 ppm, and would therefore be desirable for prospective functional electrical devices. Bai et al. [125] recorded a simple, fast and efficient method of synthesizing the PPy-rGO hybrid with oxidative chemical polymerization and loaded the hybrid with flexible PET film to develop an innovative ammonia detector. The built-in sensor not only has a 2.5-fold higher response but also shows significant selectivity at 30 °C for certain VOCs. In addition to the versatile, affordable, and compact characteristics of the hybrid-based device, the research would shed more light for the development of a type of smart wearable system. Hung et al. [126] have introduced a basic hydrothermal approach for synthesizing high quality rGO/WO_3_ nanocomposites for gas sensing applications. Gas sensing tests have shown that the nanocomposite is capable of detecting NH_3_ with a detection limit of 138 ppb at relatively low concentrations and is focused on the heterojunction between electrical conductor and the enhancement of gaseous adsorbent surface to optimize sensing efficiency. Chen et al. [127] used solvent thermal process to prepare Cu_12_Sb_4_S_13_ quantum dots@rGO nanosheet composites. With a small concentrations limit of 1 ppm, the gas sensing reaction of the nanosheet composites against ammonia is dramatically improved and it may be a successful candidate for practical detection of ammonia. In the study, nanocomposite films were constructed [128] by integrating of rGO into the PANI matrix. The responsiveness of the PANI-rGO NH_3_ gas sensors was shown to be of equal value at varying humidity levels, allowing the sensors to function in extremely humid conditions and humans to exhale in gas containing conditions. Wongchoosuk et al. [129] have been successfully fabricated the flexible Poly(3,4–ethylenedioxythiophene):poly(styrenesulfonate) gas sensors using ink-jet printing technique. The flexible graphene–PEDOT: PSS gas sensor has high sensing efficiency at room temperature up to NH_3_ with varying concentrations from 25 to 1000 ppm. In addition, the response of the gas increases dramatically with increased bending angle. The suggested approach provides many unique advantages from the findings compared to another methodologies, involving smart sensing characteristics, low level thermal operation, energy accuracy, small size, and low cost. It would also be useful for the production of future sensor applications. Yu et al. [130] proposed and demonstrated a Pt nanoparticle-assimilated GO nanocomposite-based microfiber sensor for ammonia sensing. The designed sensor has specific characteristics like the 10.2 pm/ppm responsiveness, sluggish, electromagnetic tolerance, and easy designing which make this fiber-optic sensor very auspicious for a number of sensor applications. The unique ammonia sensors, based on Pd/SnO_2_/RGO ternary nanocomposite films, were successfully fabricated using a one-pot method [131]. Authors observed that the introduction of reduced graphene oxide and palladium nanoparticles stimulated the response of the gas sensor, particularly at low quantities. Furthermore, ternary nanocomposite film doped with 0.1 g of reduced graphene oxide had an excellent accuracy even at low NH_3_ test concentration, good linearity between 5 and 150 ppm, good reversibility, fast reaction time, short recovery time, and long lasting stability at ambient temperature. For the first time Achary et al. [132] highlighted the use of rGO-CuFe_2_O_4_thatdepicted remarkable sensitivity (25% for 200ppm and 2% for 5ppm) for a broader range of NH_3_ gas, among other gases at surrounding temperature with rapid reaction and recovery periods. Their results are favorable for the design of nanocomposites based on graphene oxide for sensing applications in the material world. ZnO NW-RGO nanocomposites, which have a very tiny size and low power dissipation have been prepared by Yang et al. [133] and are essential for system integration and portable devices. Moreover, this type of sensor is expected to have a common forum for the potential identification of toxic gases. Interestingly, Huang et al. [134] analyzed the ammonia sensing performance of aniline reduced based on acid-doped CRG, de-doped CRG and completely free CRG. With an NH_3_ concentration of 50 ppm and the maximum response to 50 ppm of NH_3_, the free CRG based sensors had a 37.1% reaction but it did not return to its original value. As the way these sensors are fabricated is very basic, it is interesting and holds tremendous potential for implementations in the real world.

## 4. Conclusions

In summary, the significant progress has been made in the impressive development of mechanism designing, various composite modifications and its promising results, which could be used as the most promising candidate for the sensing of different analytes. Most of the papers have highlighted the high performance of sensor properties with different electrolytes. In this article, we focused on different types of nanostructured morphological composite materials, which showed high performance for both sensors and electrochemical sensors. The current and future perspectives of electrochemical properties of graphene and perovskite supported composites in certain sensor applications are also discussed in detail. Additionally, the new durable, simple manufacturing, lower cost, and environmentally friendly natured composite will have undoubtedly broad sensors application perspectives.

## Figures and Tables

**Table 1:** Comparison of the analytical performances based on graphene and perovskite composite and the previously reported sensing systems.

Materials	Methods	Analytes	Detection Techniques	LOD (μM)	Sensitivity (μA mM^−1^ cm^−2^)	Linear Range (μM)	Ref.
Sr_0.85_Ce_0.15_FeO_3_	Combustion	H_2_O_2_	Amperometry	10	60	-	[3]
^a^ P-L-Trp/GN	Electrochemical	Dopamine	^c^ DPV	0.06	-	0.2–100	[4]
LaTiO_3_-Ag_0.2_	Sol–gel	Glucose	Amperometry	21	784.14	2.5–400	[36]
LaNi_0.6_Co_0.4_O_3_ (LNC)	Sol–gel	Glucose	Amperometry	800	643	0.05–200	[37]
LaNi_0.5_Ti_0.5_O_3_ (LNT)	Chemical	Glucose	Amperometry	0.07	1630.57	0.2–20	[38]
GC/LaCoO_3_	Microwave-assisted	Hydrazine	Amperometry	15	561	100–15,000	[49]
rGO/CuO	Chemical	Hydrazine	Amperometry	9.8	3.87	1–400	[51]
NaNbO_3_	Solvothermal	Dopamine	DPV	680	99	10–500	[56]
RGO/ZIF-8	In situ	Dopamine	DPV	38	-	0.1–10	[62]
RGO-PtNPs	Electrochemical	H_2_O_2_	Amperometry	0.2	459±3	0.5–3.475	[64]
^b^ Co_3_O_4_-HNS/G	Self-cross link	H_2_S	^d^ GC	0.1	-	-	[90]
Pd/SnO_2_/RGO	One-pot route	NH_3_	^d^ GC	5.0	--	5–150	[131]

^a^ Polymerized L-Tryptophane; ^b^ Cobalt hallow nanosphere composite; ^c^ Differential pulse voltammetry; ^d^ Gas Chromotography.
